# Semaphorin3B promotes an anti-inflammatory and pro-resolving phenotype in macrophages from rheumatoid arthritis patients in a MerTK-dependent manner

**DOI:** 10.3389/fimmu.2023.1268144

**Published:** 2024-01-12

**Authors:** Sara Martínez-Ramos, Carlos Rafael-Vidal, Beatriz Malvar-Fernández, Nair Pérez, Coral Mouriño, Sara García Pérez, Francisco J. Maceiras Pan, Carmen Conde, Jose María Pego-Reigosa, Samuel García

**Affiliations:** ^1^Rheumatology and Immuno-mediated Diseases Research Group (IRIDIS), Galicia Sur Health Research Institute (IIS Galicia Sur), SERGAS-UVIGO, Vigo, Spain; ^2^Rheumatology Department, University Hospital Complex of Vigo, Vigo, Spain; ^3^Laboratorio de Reumatologia Experimental y Observacional y Servicio de Reumatologia, Instituto de Investigacion Sanitaria de Santiago (IDIS), Hospital Clinico Universitario de Santiago de Compostela (CHUS), Servizo Galego de Saude (SERGAS), Santiago de Compostela, Spain

**Keywords:** rheumatoid arthritis, Semaphorin3B, macrophages, inflammation, MERTK

## Abstract

Previous works from our group show that Semaphorin3B (Sema3B) is reduced in RA and plays a protective role in a mouse arthritis model. In turn, MerTK plays a protective function in murine arthritis models, is expressed by synovial tissue macrophages and is linked to remission in patients with RA. In this study, we examined the role of Sema3B in the phenotypic characteristics of RA macrophages and the implication of MerTK. Peripheral blood monocytes from RA patients were differentiated into IFN-γ (RA MØ_IFN-γ_) or M-CSF (RA MØ_M-CSF_) macrophages and stimulated with LPS, Sema3B or their combination. Alternatively, RA fibroblast like synoviocytes (FLS) were stimulated with RA MØ_IFN-γ_ and RA MØ_M-CSF_ supernatants. Gene expression was determined by qPCR and protein expression and activation by flow cytometry, ELISA and western blot. Sema3B down-regulated the expression of pro-inflammatory mediators, in both RA MØ_IFN-γ_ and RA MØ_M-CSF_. We observed a similar reduction in RA FLS stimulated with the supernatant of Sema3B-treated RA MØ_IFN-γ_ and RA MØ_M-CSF_. Sema3B also modulated cell surface markers in macrophages towards an anti-inflammatory phenotype. Besides, MerTK expression and activation was up-regulated by Sema3B, just as GAS6 expression, Resolvin D1 secretion and the phagocytic activity of macrophages. Importantly, the inhibition of MerTK and neuropilins 1 and 2 abrogated the anti-inflammatory effect of Sema3B. Our data demonstrate that Sema3B modulates the macrophage characteristics in RA, inducing a skewing towards an anti-inflammatory/pro-resolving phenotype in a MerTK-dependant manner. Therefore, here we identify a new mechanism supporting the protective role of Sema3B in RA pathogenesis.

## 1 Introduction

Rheumatoid arthritis (RA) is a chronic autoimmune rheumatic and musculoskeletal disease (RMD) characterized by articular inflammation, bone erosion and cartilage destruction. Despite the advances in the last decades, current therapies only reach persistent responses in 30% of the patients (1).

Recent studies from our group have reported the relevance of Semaphorin3B (Sema3B) in the pathogenesis of RA. On one hand, Sema3B levels are reduced in the synovial tissue and serum of RA patients compared to arthralgia and undifferentiated arthritis patients, and these levels decrease during the progression of the disease. On the other hand, Sema3B reduces migration, invasion and the secretion of matrix metalloproteases (MMPs) in RA fibroblast-like synoviocytes (FLS). More importantly, Sema3B deficiency enhances the severity of serum-induced arthritis, while Sema3B administration abrogates this effect. This protective role is associated with a reduced mouse FLS migration and the expression of inflammatory mediators in the affected joints. We also found a reduced expression of the macrophage marker CD68, suggesting that Sema3B may modulate this cell population (2–4).

Macrophages are key mediators in RA and are involved in several pathogenic processes, including inflammation, angiogenesis and bone and cartilage destruction (5, 6) importantly, the number of synovial macrophages correlates with the clinical disease activity (7). Historically, macrophages have been classified as pro-inflammatory (M1) or wound healing/anti-inflammatory (M2) macrophages, although these are the extremes of a broad spectrum of intermediated states that depend on the environmental factors and the surrounding cells (5). Recent studies have reported the existence of several macrophage subsets in the synovium of RA patients with different functional roles. Notably, the frequency of these populations is also associated with the clinical status of the patients (8, 9).

Since the effect of Sema3B on RA macrophages is unknown, in the current study we examined the effect of Sema3B on the functional and phenotypic characteristics of this cell population.

## 2 Materials and methods

### 2.1 Patients

Peripheral blood mononuclear cells (PBMCs) (n = 33) and FLS (n = 4) were obtained from blood and inflamed joints of RA patients, respectively. All subjects provided written informed consent, and the protocols were approved by the Ethics Committee of Galicia prior to patient inclusion in this study (studies numbers 2020/159 and 2021/03). RA patients fulfilled the American College of Rheumatology/European Alliance of Associations for Rheumatology 2010 classification criteria for RA (10). Clinical characteristics of patients are detailed in **Supplementary Table S1**.

### 2.2 Monocyte purification, macrophage differentiation and stimulation

PBMCs were obtained by Ficoll gradient (STEMCELL Technologies) and CD14^+^ monocytes were isolated by using the MagniSort Human pan-Monocyte isolation kit (Thermo Fisher Scientific). Monocytes were differentiated into RA macrophages (RA MØ) by culturing in Iscove’s Modified Dulbecco’s Medium (IMDM) supplemented with 10% of heat-inactivated fetal bovine serum (FBS, Corning™) and 10000 I.E penicillin-streptomycin (Lonza™ BioWhittaker™), in the presence of IFN-γ (10 ng/mL; R&D Systems; RA MØ_IFN-γ_) or M-CSF (25 ng/mL; PeproTech; RA MØ_M-CSF_) for 7 days. On one side, macrophages were differentiated in the presence or the absence of recombinant human (rh)Sema3B (200 ng/mL; R&D Systems) for 7 days. Conversely, RA MØ_IFN-γ_ and RA MØ_M-CSF_ were cultured for 7 days and stimulated for 24 h with LPS (10 ng/mL; InvivoGen) in the presence or absence of rhSema3B (200 ng/mL). RA MØ_IFN-γ_ and RA MØ_M-CSF_ were pre-incubated during 1 hour with neutralizing anti-Neuropilin-1 (α-NRP1) antibody (5µg/mL; R&D Systems), neutralizing anti-Neuropilin-2 (α-NRP2) antibody (5µg/mL; R&D Systems) or their respective isotype controls (sheep and goat IgG; 5µg/mL; R&D Systems), and stimulated with LPS (10 ng/mL) in the presence or absence of rhSema3B (200 ng/mL) for 24 h.

Alternatively, RA MØ_IFN-γ_ and RA MØ_M-CSF_ were also pre-incubated during 1 hour with DMSO or a specific MerTK inhibitor (100nM; UNC2881, Cayman Chemical) (11, 12) and stimulated with LPS in the presence or absence of rhSema3B for 24 h.

Cells were lysed for mRNA expression analysis or typsinized for Flow Cytometry. Cell-free supernatants were harvested for cytokine analysis.

### 2.3 RA FLS culture and stimulation

RA FLS were cultured in Dulbecco’s Modified Eagle Medium (DMEM, Lonza™ BioWhittaker™) containing 10% FBS, 200 mM Glutamine (Lonza™) and 10,000 U/mL penicillin-streptomycin (Thermo Fisher Scientific) and used between passages 6 to 10. Prior to stimulation, RA FLS were cultured overnight in DMEM containing 1% FBS. Afterwards, the cells were stimulated for 4 h with the supernatants (20%, v/v) from RA MØ_IFN-γ_ and RA MØ_M-CSF._

### 2.4 RT-PCR and quantitative (q)PCR

RNA from RA MØ and RA FLS was isolated employing the NucleoSpin RNA/Protein Mini kit (Macherey-Nagel). Total RNA was reverse-transcribed using iScript (Biorad). cDNA was amplified by qPCRs in duplicates using SYBR green (Biorad) and specific primers (Integrated DNA Technologies (IDT); **Supplementary Table S2**) with a CFX96 Touch Real-Time PCR Detection System (Biorad). Relative levels of gene expression were normalized to the expression of 2 housekeeping genes (*GAPDH* and *B2M*). The relative quantity (RQ) of mRNA was calculated by using the formula 2^–ΔΔCt^.

### 2.5 ELISA

IL-12p70, IL-6, TNF (R&D Systems) and Resolvin D1 (Cayman Chemical) protein levels were measured by ELISA in cell-free supernatants, according to the manufacturing instructions.

### 2.6 Flow cytometry

Data were acquired on a CytoFLEX S analyser (Beckman Coulter). 10% of Anti-Hu Fc Receptor (Thermo Fisher Scientific) was used for avoiding non-specific binding. Macrophages were stained with Fixable Viability Dye eFluor for dead cell exclusion (e450; Thermo Fisher Scientific) and antibodies for CD14-PerCPCy5.5, CD64-FITC, CD80-BV510, CD86-PECy7, CD163-APC-Cy7, CD206-APC, HLA-DR-BV605 and MerTK-PE (all Biolegend). In the case of MerTK, Fluorescence Minus One (FMO) enabled the tagging of the positive population. After excluding debris, doublets and dead cells, cell populations were analyzed using CytExpert software and Cytobank platform (Beckman Coulter). Results were expressed as the Median Fluorescence Intensity (MFI).

### 2.7 Phagocytosis assay

Macrophage phagocytic activity was determined by the uptake of the fluorogenic substrate DQ Red BSA (FITC; Thermo Fisher Scientific). Stimulated RA MØ_IFN-γ_ and RA MØ_M-CSF_ were typsinized and plated in conical well plates. DQ Red BSA (20 ng/mL) was added for 0, 30 and 60 minutes. Uptake data were acquired on a CytoFLEX S analyser (Beckman Coulter) and results were expressed as percentage of DQ-BSA uptake versus 30 minutes basal uptake.

### 2.8 Immunoblotting

Protein from RA MØ_M-CSF_ was isolated by Laemmli buffer. Equal quantities of total protein were submitted to electrophoresis on Polyacrylamide gels and transferred to PVDF Transfer membranes (Thermo Fisher Scientific). Membranes were incubated (4°C, overnight) with primary antibodies for (p)hosphoMerTK (FabGennix) and β-actin (R&D Systems) in 4% Milk-TBS/T, washed and incubated in 2% Milk-TBS/T containing HRP-conjugated secondary antibody (anti-mouse IgG, Thermo Fisher Scientific). Protein was developed with ECL Western Blotting Substrate (Thermo Fisher Scientific) employing a ChemiDocTM MP System (Biorad). Densitometry analysis was performed by ImageJ software and relative protein expression was normalized to the values for β-actin.

### 2.9 Gene expression from profiling data

The gene expression of Sema3B receptors in bone marrow-derived macrophages (BMDM) from wild type (WT) and MerTK deficient (*Mertk*^-/-^) mice was retrieved from array profiling data available at the Gene Expression Omnibus (GEO–NCBI; GSE205070) (13).

### 2.10 Statistical analyses

Statistical analysis was performed by using Windows GraphPad Prism 8 (GraphPad Software, Inc.). Normality was analysed by Shapiro-Wilk and Kolmogorov-Smirnov tests. The potential differences between experimental groups following normal distribution were analysed by One-way ANOVA and Paired t tests, as applicable. Data non-following normal distribution were analysed by Friedman’s test. P values < 0.05 were considered statistically significant.

## 3 Results

### 3.1 Sema3B modulates the inflammatory characteristics of RA MØ

**Figure 1:**
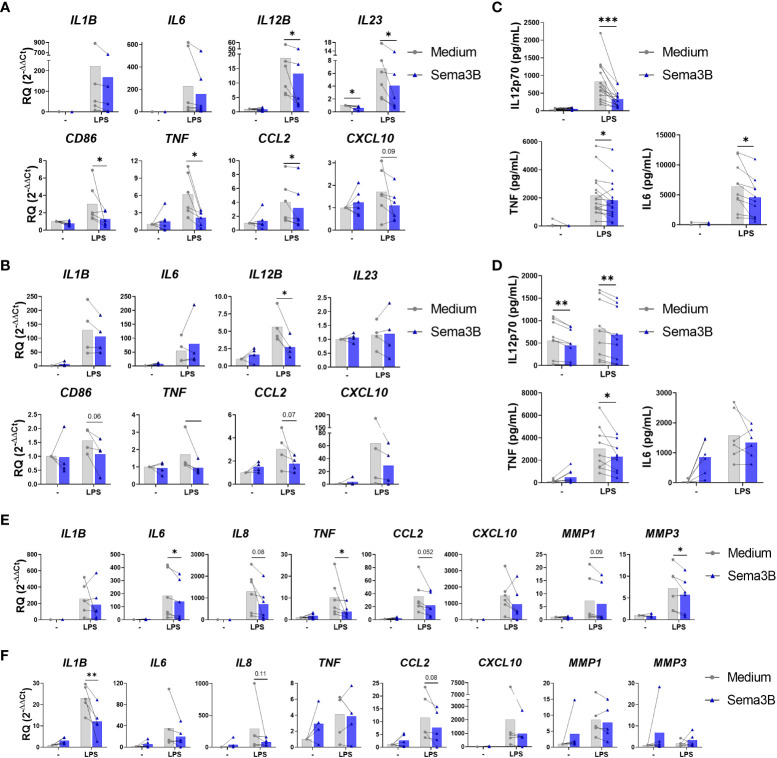
Sema3B reduces the inflammatory phenotype of RA MØ and RA FLS. **(A–D)** mRNA expression and protein secretion of inflammatory and anti-inflammatory mediators in RA MØ_IFN-γ_ [n = 6 – 16] **(A, C)** and RA MØ_M-CSF_ [n = 4 – 9] **(B, D)** stimulated with rhSema3B [200 ng/mL] in the presence or absence of LPS [10 ng/mL] for 24 h. **(E, F)** mRNA expression of inflammatory mediators in RA FLS stimulated with RA MØ_IFN-γ_ [n = 6] **(E)** and RA MØ_M-CSF_ [n = 5] **(F)** supernatants [20%, v/v] for 4 h. Data are shown as RQ (relative quantity) respect to unstimulated cells and serum concentration (pg/mL), and analysed by One-way ANOVA tests. Means and SEM are shown. *P < 0.05 and **P < 0.01 and ***P < 0.001.

We firstly analysed the effect of Sema3B in inflammatory macrophages (RA MØ_IFN-γ_). Sema3B alone did not affect the expression of pro-inflammatory mediators, but significantly reduced the LPS-induced expression of *IL12B, IL23, CD86, TNF* and *CCL2* , while moderately *IL1B *, *IL6* and *CXCL10* (**Figure 1A**). In the case of anti-inflammatory mediators, Sema3B induced the expression of *STAB1*, but did not modulate the expression of *IL10*, *IL13* and *TGFB* (**Supplementary Figure S1A**).

The influence of Sema3B was also determined on RA MØ differentiated with M-CSF, which promotes an anti-inflammatory phenotype (14). Similarly to RA MØ_IFN-γ_, Sema3B alone did not affect the expression of pro-inflammatory mediators in RA MØ_M-CSF_, but significantly down-regulated the LPS-induced expression of *IL12B* , besides reducing *CD86, TNF* and *CCL2* (**Figure 1B**). In contrast, the anti-inflammatory mediators *STAB1* and *TGFB* were up-regulated by Sema3B, alone and in combination with LPS (**Supplementary Figure S1B**).

We validated these findings at the protein level in both RA MØ_IFN_ and RA MØ_M-CSF_, in which Sema3B reduced the LPS-induced secretion of IL12p70, TNF-α and, in RA MØ_IFN-γ_ specifically, IL-6 (**Figures 1C, D**).

With the aim of determining whether the anti-inflammatory effect of Sema3B could have functional consequences on effector cells, RA FLS were stimulated with cell-free supernatants from RA MØ_IFN-γ_ and RA MØ_M-CSF_. Interestingly, supernatants of those macrophages stimulated with LPS in combination with Sema3B reduced the expression of *IL1B, IL6, IL8, TNF, CCL2, CXCL10, MMP1 and MMP3* compared to supernatants of LPS-stimulated macrophages, although differences were not significant for all mediators (**Figures 1E, F**).

**Figure 2:**
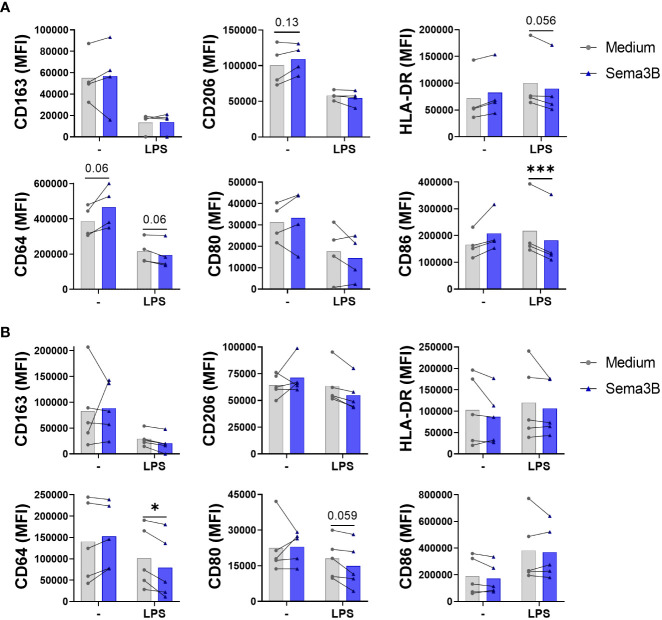
Sema3B promotes an anti-inflammatory phenotype in RA MØ. **(A, B)** CD163, CD206, HLA-DR, CD64, CD80 and CD86 cell surface marker expression in RA MØ_IFN-γ_ [n = 4] **(A)** and RA MØ_M-CSF_ [n = 5] **(B)** stimulated with rhSema3B [200 ng/mL] in the presence or absence of LPS [10 ng/mL] for 24 h. Data are shown as MFI (Median Fluorescence Intensity) of cells and analysed by Friedman **(A)** One-way ANOVA **(B)** tests. Means and SEM are shown. *P < 0.05, ***P < 0.001.

The effect of Sema3B on the modulation of M1 (CD80, CD86 and HLA-DR) and M2 (CD163 and CD206) surface markers expressed by RA synovial macrophages was also evaluated (6). In RA MØ_IFN-γ_, Sema3B alone promoted CD64 and CD206 expression and, in LPS-stimulated macrophages, reduced the expression of CD64, HLA-DR and, significatively, CD86 (**Figure 2A**). In the case of RA MØ_M-CSF_, we found that, in combination with LPS, Sema3B decreased the expression of CD64 and a trend for CD80 (**Figure 2B**). We also determined whether Sema3B was able to modulate the expression of specific markers during the macrophage differentiation process. Sema3B significantly down-regulated the expression of HLA-DR in IFN-γ-differentiated macrophages, but it did not modulate M2 markers. During the differentiation process with M-CSF, Sema3B reduced the expression of HLA-DR and CD64, while it significantly induced the expression of CD163 (**Supplementary Figure S2**).

Altogether, these data demonstrate an anti-inflammatory effect of Sema3B in RA MØ, by reducing the expression of the inflammatory phenotype and modulating the phenotypic characteristics of RA MØ.

### 3.2 Sema3B promotes an anti-inflammatory and pro-resolving macrophage phenotype in a MerTK-dependant manner

**Figure 3:**
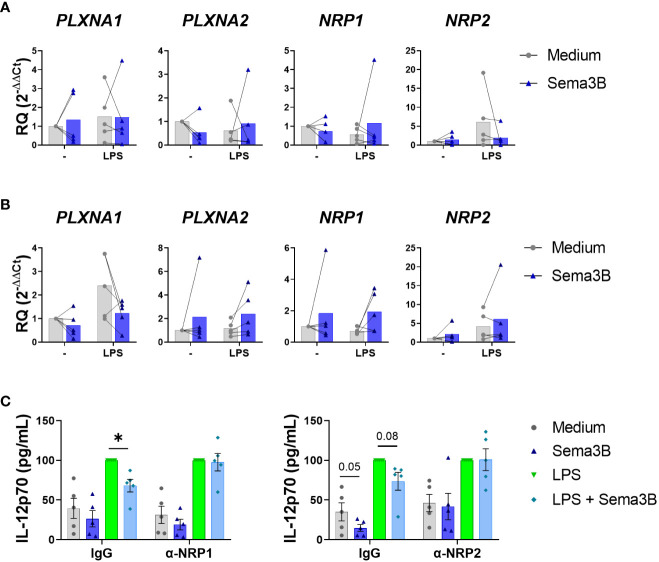
The protective effect of Sema3B in RA MØ is mediated by the co-receptors NRP1 and NRP2. **(A, B)** mRNA expression of Sema3B receptors in RA MØ_IFN-γ_ [n = 5] **(A)** and RA MØ_M-CSF_ [n = 5] **(B)** stimulated with rhSema3B [200 ng/mL] in the presence or absence of LPS [10 ng/mL] for 24 h. **(C)** IL12p70 secretion in RA MØ_IFN-γ_ [n = 5] pre-incubated with neutralizing α-NRP1, α-NRP2 antibodies [5µg/mL] or their respective isotype controls, and stimulated with LPS [10 ng/mL] in the presence or absence of rhSema3B [200 ng/mL] for 24 h. Data are shown as RQ (relative quantity) respect to unstimulated cells and serum concentration (pg/mL) and analysed by One-way ANOVA tests. Means and SEM are shown. *P < 0.05.

We next sought the mechanisms involved in the anti-inflammatory effect of Sema3B. Sema3B did not modulate the expression of its known receptors, PlexinA1 and PlexinA2, and co-receptors, Neuropilin-1 (NRP-1) and Neurolipin-2 (NRP-2) in either RA MØ_IFN-γ_ or RA MØ_M-CSF_ (15, 16) (**Figures 3A, B**). However, the neutralization of both NRP-1 and NRP-2 abolished the Sema3B-mediated decrease of IL12p70 in RA MØ_IFN-γ,_ demonstrating that both Sema3B co-receptors are involved in the anti-inflammatory role of Sema3B (**Figure 3C**).

**Figure 4:**
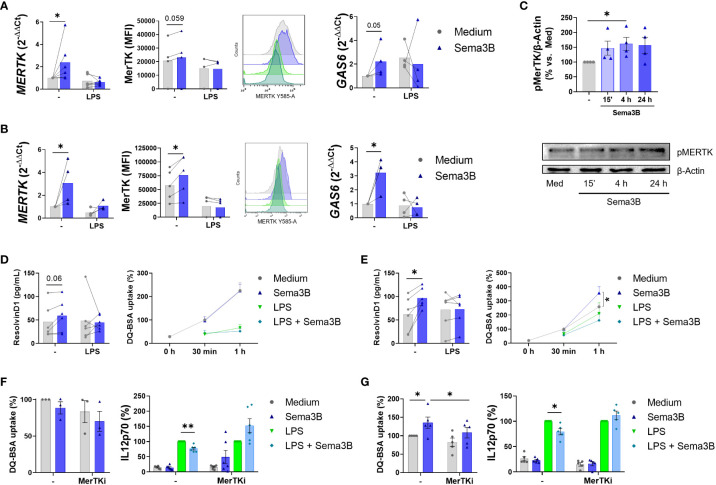
Sema3B induces an anti-inflammatory/pro-resolving phenotype in RA MØ via MerTK. **(A, B)** MerTK and GAS6 mRNA levels and MerTK cell surface expression in RA MØ_IFN-γ_ [n = 4 – 6] **(A)** and RA MØ_M-CSF_ [n = 4 – 5] **(B)** stimulated with Sema3B [200 ng/mL] in the presence or absence of LPS [10 ng/mL] for 24 h. **(C)** Densitometric analysis and representative immunoblot of MertK activation ((p)hosphoMerTK) in RA MØ_M-CSF_ [n = 4] stimulated with rhSema3B [200 ng/mL] for 15 minutes, 4 and 24 hours. **(D, E)** Resolvin D1 secretion and phagocytic activity in RA MØ_IFN-γ_ [n = 4 – 6] **(D)** and RA MØ_M-CSF_ [n = 5 – 6] **(E)** after stimulation with rhSema3B [200 ng/mL] in the presence or absence of LPS [10 ng/mL] for 24 h. **(F, G)** Phagocytic activity and IL12p70 secretion in RA MØ_IFN-γ_ [n = 3 – 6] **(F)** and RA MØ_M-CSF_ [n = 5] **(G)** pre-incubated with the MerTKi (i)nhibitor UNC2881 [100 nM] and stimulated with rhSema3B [200 ng/mL] in the presence or absence of LPS [10 ng/mL] for 24 h. Data are shown as RQ (relative quantity) respect to unstimulated cells, MFI (Median Fluorescence Intensity) of cells, % of pMerTK compared to medium, serum concentration (pg/mL), % of DQ-BSA uptake compared to medium and % of IL12p70 secretion compared to LPS. Data are analysed by One-way ANOVA tests. Means and SEM are shown. *P < 0.05, **P < 0.01.

Due to the crucial role of MerTK in resolving inflammation in RA (9, 17, 18), the involvement of Sema3B on the expression of this tyrosine kinase receptor was also evaluated. Sema3B alone, but not in combination with LPS, up-regulated the mRNA and protein expression of MerTK, and the mRNA expression of the MerTK ligand GAS6 (19–21) in both RA MØ_IFN-γ_ (**Figure 4A**) and RA MØ_M-CSF_ (**Figure 4B**), although differences were more pronounced in the latter. Sema3B also increased MerTK expression during the differentiation process into RA MØ_M-CSF_ (**Supplementary Figure S2B**). Importantly, Sema3B induced the activation of MerTK in RA MØ_M-CSF_ at different time points, being this effect significant at 4 hours (**Figure 4C**).

Since MerTK^+^ RA MØ are involved in the resolution of inflammation, we next analysed the effect of Sema3B in the phagocytic activity of macrophages and the synthesis of Resolvin D1, a lipid induced by macrophage MerTK signalling involved in resolving joint inflammation (22). In RA MØ_IFN-γ_, Sema3B moderately increased Resolvin D1 secretion, although it did not modulate the macrophage phagocytic activity (**Figure 4D**). On their part, Sema3B-stimulated RA MØ_M-CSF_ significantly raised the secretion of Resolvin D1, as well as their phagocytosis capacity, although it resulted insufficient counteracting the effect of LPS (**Figure 4E**).

Ultimately, we tested if these responses were mediated by MerTK, using a specific MerTK (i)nhibitor. Sema3B reduced the LPS-mediated secretion of IL12p70 in both RA MØ_IFN-γ_ and RA MØ_M-CSF,_ and this effect was abrogated in the presence of the MerTKi (**Figures 4F, G**). Also, MerTK inhibition reversed the Sema3B-induced phagocytic activity of RA MØ_M-CSF_ (**Figure 4G**).

Therefore, these results suggest that the anti-inflammatory and pro-resolving responses induced by Sema3B are, at least in part, mediated by MerTK.

## 4 Discussion

In this manuscript we demonstrate that Sema3B modulates the phenotypic characteristics of RA macrophages. More specifically, Sema3B induces a skewing towards an anti-inflammatory/pro-resolving phenotype in a MerTK-dependant manner. Through this work we have found a new protective effect of Sema3B in the pathogenesis of RA. This protective role is in line with previous results from our group, in which we described a reduced expression of inflammatory mediators in both joints and FLS of arthritic mice treated with Sema3B and an impaired invasive phenotype in Sema3B-stimulated RA FLS (2–4).

Firstly, Sema3B reduced the secretion of pro-inflammatory cytokines in monocytes-derived macrophages from RA patients. The expression of surface markers associated to M1 macrophages (CD86, HLA-DR and CD64) was also decreased by Sema3B, while the expression of M2 markers (Stabilin-1 and TGF-β) was raised. Neutralization experiments showed that co-receptors NRP-1 and NRP-2 are involved in the protective signalling induced by Sema3B. Since our previous findings identified NRP-2 and, in lower extent NRP-1, as Sema3B co-receptors essential for reducing the invasive ability of RA FLS (2), we demonstrate that Sema3B signals through these co-receptors in both cell types. Sema3B also increased the phagocytic activity in RA MØ_M-CSF_, which is linked to the resolution of tissue inflammation (6). However, we did not observe this effect in RA MØ_IFN-γ._ This might be due to the IFN-γ-induced M1-like phenotype, which could be modulated to a less inflammatory phenotype by Sema3B, but without reaching pro-resolving characteristics, as it would be the case for Sema3B-modulated RA MØ_M-CSF_. In this regard, the higher phagocytic activity of RA MØ_M-CSF_ compared to RA MØ_IFN-γ_ (**Supplementary Figure S3**), and the lack of effect of Sema3B on LPS-stimulated macrophages, support this aim.

Secondly, we proved that the anti-inflammatory/resolving effect of Sema3B in RA MØ was mediated by MerTK. Sema3B has a dual role on MerTK activation. On one hand, Sema3B induced the expression and activation of MerTK. On the other hand, Sema3B up-regulated the macrophage expression of GAS6, which is a ligand that activates MerTK signalling (19–21). MerTK is a crucial tyrosine kinase for the macrophage differentiation towards an anti-inflammatory/resolving phenotype (23, 24) and its deficiency exacerbates the severity of collagen-induced arthritis, while MerTK signalling activation reduces it in this arthritis model (17, 18). Importantly, MerTK^+^ synovial macrophages are associated with the disease status of RA patients. In fact, the percentage of this macrophage population is reduced in patients with active RA compared to patients in remission and negatively correlates with the disease activity score-28 (DAS28). In contrast, MerTK^-^ synovial MØ express inflammatory mediators, induce the production of inflammatory mediators by RA FLS and participate in both bone and cartilage destruction (9, 17). Therefore, the modulation of MerTK^+^ macrophages by Sema3B may be a useful approach for the treatment of RA.

We cannot rule out the possibility that MertK modulates Sema3B signalling, since MerTK regulates the expression of the Sema3B receptor PlexinA1 (25). We analysed, using a public dataset (GSE205070), the expression of Sema3B receptors in bone marrow-derived macrophages (BMDM) from WT and *Mertk*^-/-^ mice. Deficiency of MerTK significantly up-regulated the expression of *Plxna1* and *Nrp1* (**Supplementary Figure 4**), suggesting that MerTK signalling might modulate the Sema3B signalling. However, further studies are needed for elucidating this effect.

Sema3B is not the only class 3 semaphorin member able to modulate the macrophage phenotype characteristic. In fact, Sema3A and Sema3E also induce a skewing towards a resolving/anti-inflammatory phenotype in this cell type (26–29). In addition, Sema3A and Sema3F play protective roles in the pathogenesis of RA (2, 4, 29, 30). However, these semaphorins did not modulate the expression of MerTK (data non shown), suggesting that the MerTK regulation is specific for Sema3B.

Lastly, Sema3B also enhanced the secretion of Resolvin D1, a pro-resolving lipid triggered by the MerTK signalling activation, with a protective function in inflammatory arthritis (9, 22). Moreover, Resolvin D1 has been also involved in the modulation of macrophage polarization and in the skewing towards the anti-inflammatory/resolving phenotype (20, 31).

A limitation of this study is that we did not used liquid chromatography–mass spectrometry, the gold standard for the assessment of Resolvin D1 (32). Instead, we employed the well-validated ELISA technique. We neither utilized synovial macrophages from RA patients. We used instead *in vitro* M-CSF- and IFN-γ- differentiated macrophages from peripheral blood monocytes of RA patients, which possess anti-inflammatory/pro-resolving and pro-inflammatory characteristics, respectively (5, 14). Remarkably, MerTK expression was higher in RA MØ_M-CSF_ than in RA MØ_IFN-γ_, therefore these macrophage phenotypes partially mimic the phenotypes of MerTK^+^ and MerTK^-^ synovial macrophages.

Altogether, our work identifies a new anti-inflammatory mechanism of Sema3B, confirming the protective role of Sema3B in RA pathogenesis and pointing out this semaphorin as a promising therapeutic target.

## Data availability statement

The raw data supporting the conclusions of this article will be made available by the authors, without undue reservation.

## Ethics statement

The studies involving humans were approved by Ethics Committee of Galicia (studies numbers 2020/159 and 2021/03). The studies were conducted in accordance with the local legislation and institutional requirements. The participants provided their written informed consent to participate in this study.

## Author contributions

SM-R: Conceptualization, Data curation, Formal analysis, Investigation, Methodology, Writing – original draft, Writing – review & editing. CR-V: Data curation, Formal analysis, Investigation, Methodology, Writing – review & editing. BM-F: Data curation, Formal analysis, Investigation, Methodology, Writing – review & editing. NP: Data curation, Investigation, Methodology, Writing – review & editing. CM: Data curation, Investigation, Methodology, Writing – review & editing. SGP: Data curation, Investigation, Methodology, Writing – review & editing. FJMP: Data curation, Investigation, Methodology, Writing – review & editing. CC: Conceptualization, Formal analysis, Writing – review & editing. JP-R: Formal analysis, Writing – review & editing. SG: Conceptualization, Data curation, Formal analysis, Funding acquisition, Investigation, Methodology, Project administration, Resources, Supervision, Writing – original draft, Writing – review & editing.
