# Supplementary material for: Semaphorin3B promotes an anti-inflammatory and pro-resolving phenotype in macrophages from rheumatoid arthritis patients in a MerTK-dependent manner
Source: Front Immunol. 2024 Jan 12;14:1268144. doi: 10.3389/fimmu.2023.1268144 (PMC10811190; doi:10.3389/fimmu.2023.1268144)
Supplement: Supplementary file 1 [file DataSheet_1.pdf]

Supplementary figures

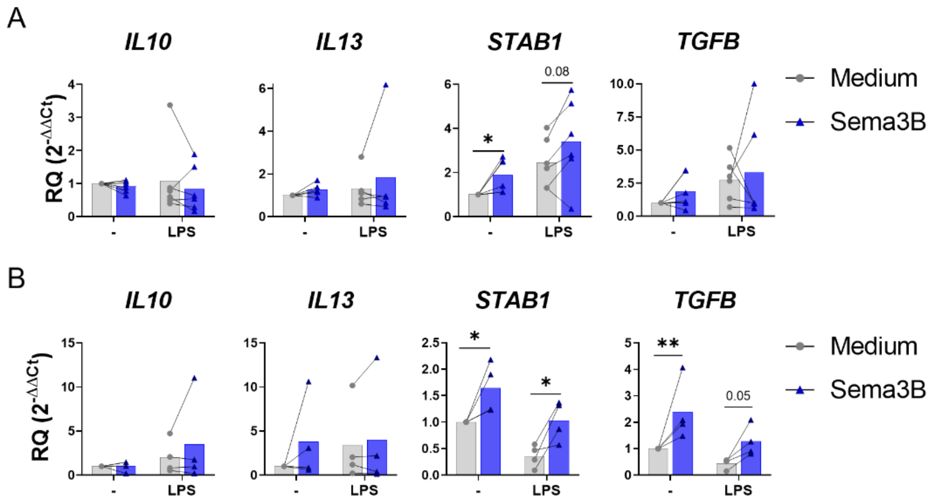

Supplementary Figure S1. Sema3B induces the expression of anti-inflammatory mediators in RA-MØ (A-B). mRNA expression of anti-inflammatory mediators in RA-$MØ_{IFN}$ [n = 5] (A) and RA-$MØ_{M-CSF}$ [n = 4] (B) stimulated with rhSema3B [200 ng/mL] in the presence or absence of LPS [10 ng/mL] for 24 h. Data are shown as RQ (relative quantity) respect to unstimulated cells and analysed by One-way ANOVA tests. Means and SEM are shown. *P < 0.05 and **P < 0.01.

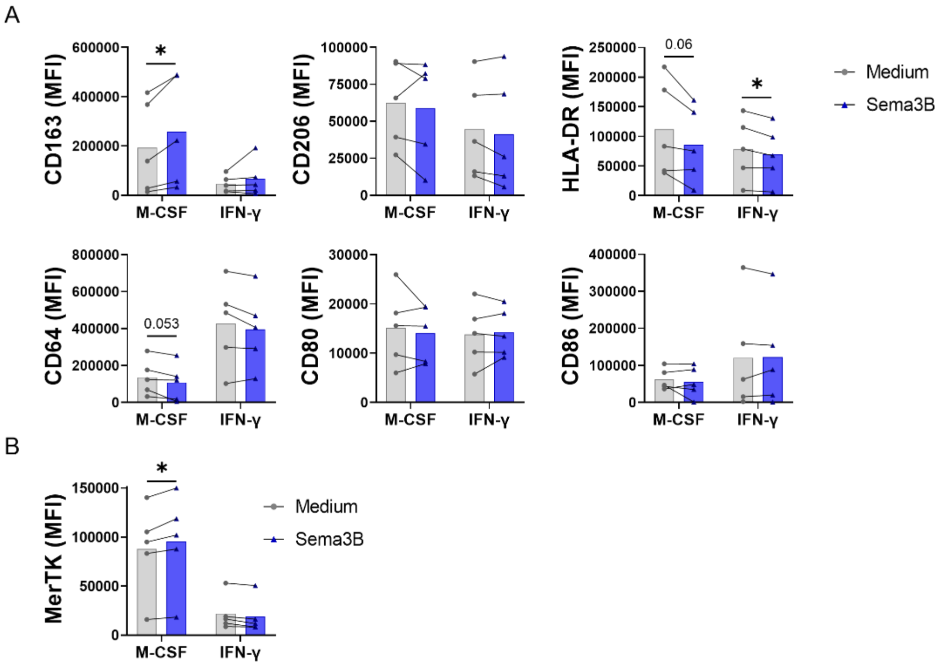

Supplementary Figure S2. Sema3B modulates RA-MØ towards an anti-inflammatory phenotype (A, B). CD64, CD80, CD86, HLA-DR, CD163, CD206 (A) and MerTK (B) cell surface marker expression in monocytes differentiated into macrophages for 7 days with IFNγ [10 ng/mL] [n = 5] or M-CSF [25 ng/mL] [n = 5] in the presence or absence of Sema3B [200 ng/mL]. Data are shown as MFI (Median Fluorescence Intensity) of cells and analysed by Paired t tests. Means and SEM are shown. *P ≤ 0.05.

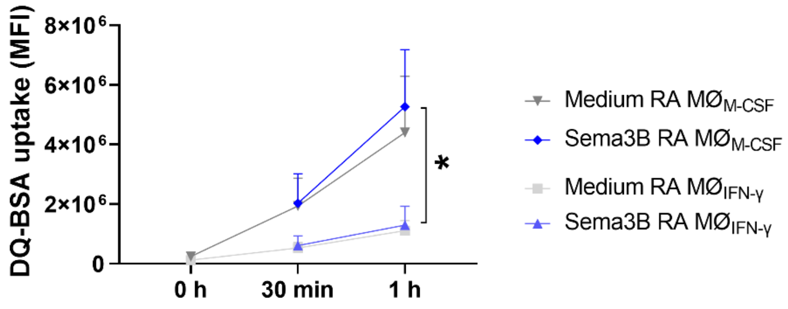

Supplementary Figure S3. M-CSF induces phagocytosis in RA-MØ. Phagocytic activity of RA-MØ$_{IFN}$ [n = 4] and RA-MØ$_{M\text{-}CSF}$ [n = 4]. Data are shown as MFI (Median Fluorescence Intensity) of DQ-BSA uptake and analysed by One-way ANOVA tests. Means and SEM are shown. *P ≤ 0.05.

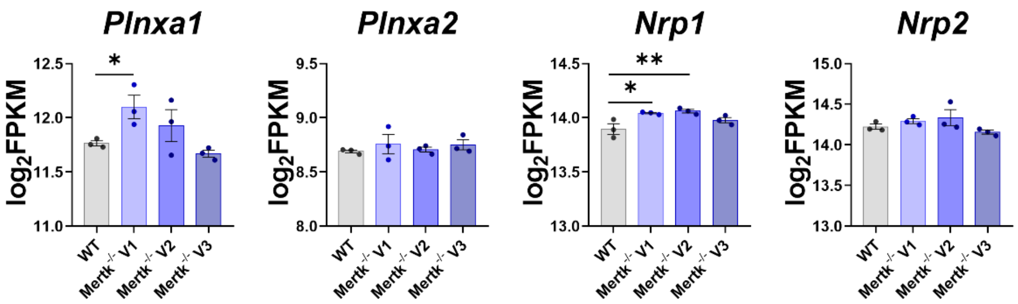

Supplementary Figure S4. MerTK signalling modulates the Sema3B receptors and co-receptors. Expression of Sema3B receptors and co-receptors in BMDM from *Mertk*$^{-/-}$ mice [n = 3]. Data are analysed by One-way ANOVA tests. Means and SEM are shown. *P < 0.05, **P < 0.01, ****P < 0.0001.

Supplementary tables

	RA (n = 33)
**Age (years)**	53.5 (45 – 69.3)
**Female: n (%)**	28 (84.8 %)
**RF$^+$**	30 (90.9 %)
**ACPA$^+$**	29 (87.9 %)
**ESR (mm/Hr)**	26 (17.0 – 46.0)
**CRP (mg/L)**	< 4.0 (< 4.0 – 10.3)
**DAS28**	3.6 (2.9 – 4.1)
**NSAIDs**	10 (30.3 %)
**DMARDs**	26 (78.8 %)
**Biologicals**	9 (27.3 %)

Supplementary Table S1. Characteristics of RA patients at the time of blood sampling. Data is presented as the median (interquartile range) or number (percentage). RF: rheumatoid factor; ACPA: Anti-citrullinated protein antibodies; ESR: erythrocyte sedimentation rate; CRP: C-reactive protein; DAS28: Disease Activity Score 28; NSAIDs: non-steroidal anti-inflammatory drugs; DMARDs: disease-modifying antirheumatic drugs.

Gene	Primer forward	Primer reverse
B2M	*GATGAGTATGCCTGCCGTGT*	*TGCGGCATCTTCAAACCTCC*
CCL2	*TCTGTGCCTGCTGCTCATAG*	*GGGCATTGATTGCATCTGGC*
CXCL10	*TGAAATTATTCCTGCAAGCCAA*	*CAGACATCTCTTCTCACCCTTCTTT*
GAPDH	*GCCAGCCGAGCCACATC*	*TGACCAGGCGCCCAATAC*
GAS6	*TGGCATGTGGCAGACAATCT*	*TGGTGTCTTCTCCGTTCAGC*
IL10	*GAGGCTACGGCGCTGTCAT*	*CCACGGCCTTGCTCTTGTT*
IL12B	*ACGTTTCACCTGCTGGTGGCT*	*CTCCGCACGTCACCCCTTGG*
IL13	*AACCTGACAGCTGGCATGTACTGT*	*CAGAATCCGCTCAGCATCCT*
IL1B	*ATGATGGCTTATTACAGTGGCAA*	*GTCGGAGATTCGTAGCTGGA*
IL23p19	*CAA CAG TCA GTT CTG CTT GC*	*GAA GGC TCC CCT GTG AAA AT*
IL6	*GACAGCCACTCACCTCTTCA*	*CCTCTTTGCTGCTTTCACAC*
MERTK	*CGCTCTGGCGTAGAGCTATC*	*AGGCTGGGTTGGTGAAAACA*
STAB1	*GTGCTAAAGAAGGGCTGTGC*	*TTGCTCTCCATGCTTGTTTG*
TGFB	*CTTCTCTCCAGCCGAGCTTC*	*TGTGTTTCGTGCAACCATCC*
TNF	*TCTTCTCGAACCCCGAGTGA*	*CCTCTGATGGCACCACCAG*

Supplementary Table S2. List of primers. Length data in base pairs.
